# Non-ammoniagenic proliferation and differentiation media for cultivated adipose tissue

**DOI:** 10.3389/fbioe.2023.1202165

**Published:** 2023-07-24

**Authors:** S. Hubalek, J. Melke, P. Pawlica, M. J. Post, P. Moutsatsou

**Affiliations:** ^1^ Mosa Meat BV, Maastricht, Netherlands; ^2^ Department of Physiology, Faculty of Health, Medicine and Life Sciences, Maastricht University, Maastricht, Netherlands; ^3^ CARIM, School of Cardiovascular Diseases, Faculty of Health, Medicine and Life Sciences, Maastricht University, Maastricht, Netherlands

**Keywords:** cultured meat, adipogenic medium, ammonia-free cell culture, glutamine substitution, ammonia inhibition

## Abstract

Ammonia (Amm), and its aqueous solved state, ammonium, which is produced from glutamine (Gln) metabolism, is a known inhibitor of stem cell proliferation *in vitro*. In the context of cultivated beef, primary bovine fibro-adipogenic progenitor cells (FAPs) need to be grown and differentiated for several weeks *in vitro* for the production of cultivated fat. In this study, the ammonium sensitivity of these cells was investigated by introducing ammonium chloride, which was found to inhibit their proliferation when above 5 mM and their adipogenic differentiation when above 2 mM. Novel serum-free proliferation and differentiation media were hence developed with the aim to suppress Amm production during expansion and adipogenesis. Glutamine substitutes, such as a-ketoglutarate (aKG), glutamate (Glt) and pyruvate (Pyr) were investigated. It was found that aKG based proliferation medium (PM) was the most effective in promoting and maintaining FAPs growth over several passages while the specific Amm production rate was reduced more than 5-fold. In terms of differentiation capacity, the substitution of glucose (Gluc) and Gln with galactose (Gal) and Pyr was shown to be the most effective in promoting FAPs differentiation into mature adipocytes, resulting in over 2-fold increase of fat volume per cell, while suppressing Amm production. Our findings suggest that FAPs do not require Gln as an essential nutrient but, on the contrary, possess all the necessary metabolic pathways to proliferate and subsequently differentiate in a Gln-free medium, resulting in decreased Amm production rates and seemingly synthesising glutamine *de novo*. These findings are important for prolonging the lifespan of culture medium, allowing for reduced costs and process interventions.

## Introduction

Glucose (Gluc) and glutamine (Gln) are thought to be essential for cell growth, acting as a main source of carbon and energy for various mammalian cell types (Vriezen et al., 1997; Vergara et al., 2018). They are found at relatively high concentrations in commercial basal culture medium formulations, such as DMEM and DMEM/F12; up to 21 mM for Gluc and 4 mM for Gln. These media are commonly used to culture primary cells suitable for cultured meat and fat production, such as fibro-adipogenic progenitors (FAPs), satellite cells (SCs) and adipose derived mesenchymal stem cells (MSCs) (Mehta et al., 2019; Hanga et al., 2021; Stout et al., 2021; Dohmen et al., 2022; Kolkmann et al., 2022; Messmer et al., 2022).

However, high concentrations of Gluc and Gln are also known to promote fast accumulation of metabolic waste, while other compounds, which are typically provided at lower concentrations and are also metabolised by the cells (i.e., other amino-acids and vitamins) do not contribute as much to metabolic waste accumulation (Lim et al., 2010; Post et al., 2020).

Ammonia (Amm) and its solved ion in aqueous medium, ammonium, is a result of the metabolization of Gln by the cells or of its spontaneous degradation in the medium. Amm has been found toxic at concentrations as low as 2 mM for various cell types (Hassell et al., 1991; Schneider, 1996; Schop et al., 2009). Amm toxicity is suggested to be deriving from the competition of ammonium with potassium ions on transporter proteins, causing increased energy demand for maintenance of ion-gradients over the cytoplasmic membrane, as well as from the free diffusion of ammonium molecules across the membrane, causing an intracellular pH shift. It has also been reported to reduce metabolic efficiency by forcing excretion of potentially valuable intermediate metabolites, such as alanine, in order to achieve detoxification (Martinelle & Häggström, 1993; Schneider, 1996). For brain cells it was previously described how ammonium exposure leads to oxidative stress, energy deficiency and apoptosis by impairing the tri-carboxylic acid (TCA) cycle and opening of the mitochondrial permeability transition pore, resulting in decreased mitochondrial functionality (Braissant et al., 2013).

For cost and resource efficient production of cultured meat, high density cultures of more than 4 × 10^7^ cells·ml^-1^ in large volumes (>1000L bioreactors) are suggested (Specht, 2020). High cell density cultures deplete nutrients and accumulate Amm fast and since the frequency of medium exchanges can depend on the latter, media still containing valuable nutrients may get unnecessarily wasted (Vis et al., 2020). Therefore, waste accumulation as a parameter for medium replenishment represents a big challenge for upscaling cultured meat production, especially since pharma-grade culture medium is still expensive and represents one of the major cost drivers (Bhat et al., 2019; Stout et al., 2021; Hubalek et al., 2022; Kolkmann et al., 2022). As such, frequent replacing of the culture medium is not a cost-viable solution during cultured meat production. Medium recycling and purification have been proposed to circumvent the need for frequent medium replacement during cultured meat production, using algae (Haraguchi & Shimizu, 2021a; 2021b; Hubalek et al., 2022), however research in this field is still at nascent stage. Other methods reported for decreasing or removing Amm from liquids are ion-exchange membranes (Sikdar & Sawant, 1994), zeolites, aluminosilicate minerals (Demir et al., 2002) or synthetic resins (Ding & Sartaj, 2016). However, Amm removal would increase the number of processing steps and as a result the manufacturing facility’s footprint and CAPEX costs. It would be more efficient to tackle the Amm accumulation issue at its root and use methods which reduce or prevent the formation of Amm in the first place. Such methods are based on a metabolic approach, harnessing the innate ability of mammalian cells to shift their metabolic substrate preference and switch between oxidative phosphorylation and glycolysis. Exchanging Gln or Glutamax (GlnX) for alternative compounds participating in glutaminolysis or the TCA cycle, such as Pyr, aKG and Glt has been applied successfully to some mammalian cell lines in the past (Hassell & Butler, 1990; Christie & Butler, 1999; Genzel et al., 2008; Ha & Lee, 2014), however never on primary cells or cells relevant to cultured meat. To our knowledge, it is also the first time that such metabolic methods are applied for adipogenesis.

Pyr participates in amino acid transamination but is also an end product of glycolysis, where it holds a central role in amino acid, lipid and Gluc metabolism (Corbet, 2018). It is the main conductor of the metabolization of Gluc into lactate (Lac) and by supplying Acetyl-CoA to the TCA cycle it generates energy and maintains the TCA cycle by converting the carbon on the amino acids’ backbones to carbon dioxide (Gambhir et al., 2003) (Supplementary Figure S1). Previous research has shown that 2 mM Gln could be replaced by 10 mM Pyr for MDCK, BHK21 and CHO-K1 cells during long-term culturing, without loss of proliferative capacity and with no Amm release into the cell culture medium (Genzel et al., 2008). Another TCA cycle participant, aKG, is a metabolite of glutaminolysis and, like Pyr, it is a keto acid and therefore also plays a crucial role in fatty acid, Gluc and amino acid oxidation (S. Liu et al., 2018) (Supplementary Figure S1).

Glt, a metabolite of Gln and a precursor of aKG, consists of less nitrogen moieties than Gln, and therefore, its metabolization results in less Amm, rendering it a potentially suitable replacement of Gln (Christie & Butler, 1999). It has been shown that Glt and aKG can support growth of several cell lines (McDermott & Butler, 1993), while significantly decreasing Amm production (>60% for BHK-21 cells and up to 70% for McCoy cells) (Hassell & Butler, 1990; Christie & Butler, 1999).

To our knowledge, no studies have explored the impact of Amm on FAPs adipogenesis or have looked into using Gln substitutes for reducing the Amm production in primary cell cultures.

The aim of this study is to test if primary bovine FAPs, a cell type suitable for cultured fat production, are able to proliferate and differentiate without Gln during long-term culture so as to reduce Amm accumulation, which in turn is suggested to extend the medium lifespan.

## Materials and methods

### Cell culture

Primary FAPs were isolated from fresh bovine muscle tissue (*Bos taurus*, Belgian Blue) samples from the semitendinosus muscle of sixteen different donors (male and female, aged from 1 to 7 years) as previously described (Dohmen et al., 2022). The semitendinosus muscle was chosen because, from an anatomical point of view, it can conveniently be sampled with a biopsy or slaughterhouse strategy. Approximately 100,000 FAPs were obtained from 1 g of digested muscle tissue.

### Culture media

For FAPs proliferation studies, the serum-free/animal-free proliferation medium (PM) previously developed (Kolkmann et al., 2022) was used and was supplemented with 17 mM Gluc (G8270, Sigma-Aldrich) and 2 mM GlutaMAX™ (GlnX) (35050061, Gibco™, Thermo Fisher Scientific). GlnX is a dipeptide of Gln and alanine and is more stable in aqueous medium than Gln (Tareq et al., 2007). For testing Amm reducing substrates during proliferation, GlnX was replaced with either 10 mM aKG (K1128, Sigma-Aldrich), 10 mM Pyr (P5280, Sigma-Aldrich) or 10 mM Glt (G5889, Sigma-Aldrich).

For FAPs differentiation studies, the serum-free adipogenic differentiation as previously described (Dohmen et al., 2022; Mitić et al., 2023) was used as control differentiation medium (DM), with slight changes to the inducers (Supplementary Table S1). The basal medium was either DMEM (12320032, Gibco™) or DMEM/F12 (P04-41505, Pan Biotech) without Gluc and Gln, which was supplemented with 17 mM of Gluc and 4 mM of Gln (or GlnX). To test ammonia-reducing substrates during differentiation, GlnX was replaced with 10 mM Pyr, or both Gluc and GlnX were replaced with a combination of 17 mM galactose (Gal) (G0750, Sigma-Aldrich) and 10 mM Pyr.

### FAPs proliferation on planar systems

For planar cultures, FAPs were sub-cultured in standard tissue culture flasks, seeded at 5,000 cells/cm^2^ (50.000 cells·ml^-1^) and passaged when confluent (ca. 40.000 cells/cm^2^) using 10x TryplE^TM^ Select Enzyme for dissociation (dilution 3:10; A1217702, Gibco™) in PBS (10010023, Gibco™). Medium was exchanged at every passage. Cell counts were obtained using Trypan Blue staining (T8154, Sigma Aldrich), and counting was performed with an automated cell counter (Invitrogen™ Countess™ II FL, Thermo Fisher Scientific). Specific growth rates (*μ*) and doubling times (DTs) were calculated after each passage using total viable cell counts as follows:

$$\mu =\frac{\left(\ln\frac{X}{X_{0}}\right)}{\Delta t}$$ (1)

$$DT=\frac{\ln\left(2\right)}{\mu }$$ (2)

where, μ = specific growth rate (h^-1^), X_0_ = number of seeded cells, X = number of harvested cells, Δt = time elapsed from seeding till harvesting (h).

### FAPs proliferation on Cytodex-1 microcarriers

**FIGURE 1:**
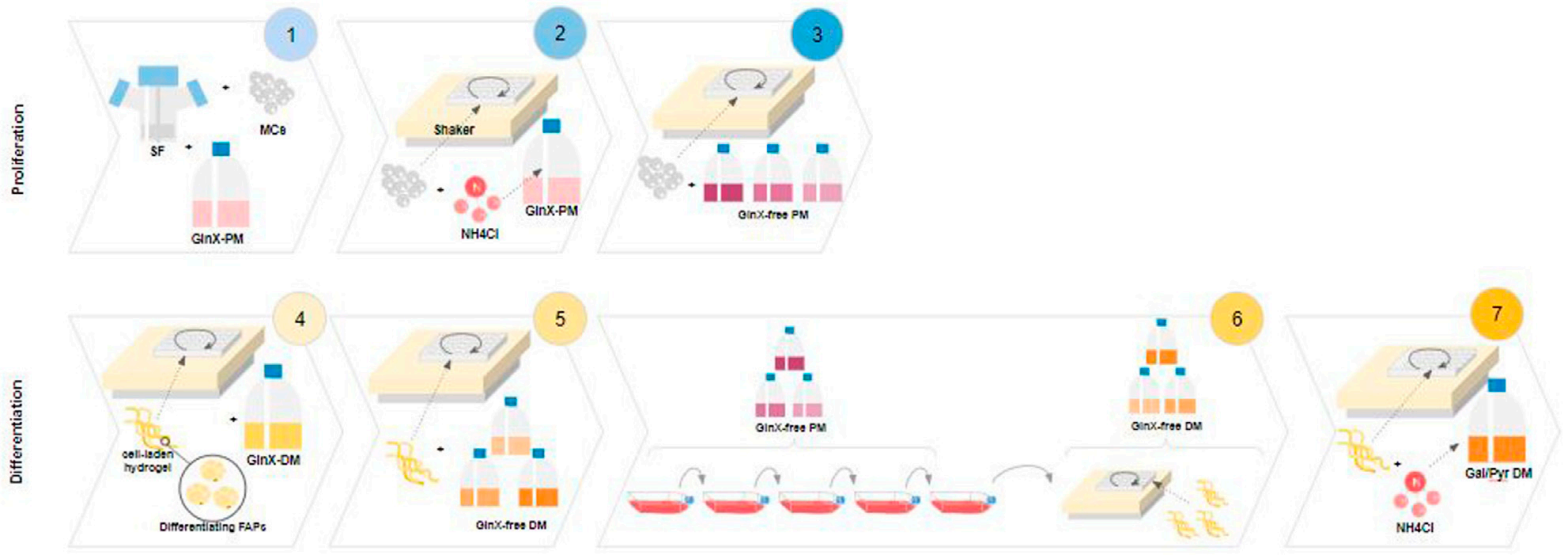
Experimental designs. 1-3 Proliferation. 4-7 Differentiation. **1**: Spinner flask proliferation of FAPs in Gluc/GlnX medium (control) and metabolite analysis; **2**: Testing Amm sensitivity during short-term proliferation on an orbital shaker setup; **3**: Screening of Amm reducing substrates during short-term proliferation in an orbital shaker setup; **4**: Hydrogel based adipogenic differentiation of FAPs in Gluc/GlnX medium and medium volume reduction; **5**: Screening of Amm reducing substrates during fat differentiation of FAPs; **6**: Long-term passaging of FAPs in Amm reducing substrates with subsequent fat differentiation; **7**: Testing Amm sensitivity during adipogenesis in Amm reducing substrates.

Cells were cultured on microcarriers in two setups: 1) 100 ml glass spinner flasks (Bellco™) with 100 ml of culture medium or 2) 24-well ultra-low attachment plates with 1 ml of medium, both with 10 cm^2^·ml^−1^ (Cytodex^®^-1, Cytiva) (Figure 1, steps 1–3). Prior to inoculation, spinner flasks were siliconized with Sigmacote^®^ (SL2, Sigma-Aldrich) and rinsed thoroughly with water. Subsequently, microcarriers were weighed into the spinner flasks, hydrated in PBS and autoclaved. After inoculation, the spinner flasks were placed on magnetic stirring platforms (Cimarec™ Biosystem; Thermo Fisher Scientific) set at 45 rpm. The well plates were placed on an orbital shaker platform (88881102B, Thermo Fisher Scientific) at 70–90 rpm. Each culture condition was split over 6 separate wells, to be able to perform a daily cell count for 6 consecutive days, by sacrificing one well per day. For both setups, daily cell counts, imaging and metabolite analyses were performed.

Cell counts were always performed on at least two independent samples with the use of Nucleocounter (NC200™, Chemometec). Briefly, a homogeneous cell/microcarrier suspension was lysed with reagent A100 (910–0003, Chemometec) and stabilised with reagent B (910–0002, Chemometec) before being loaded on a DAPI and Acridine orange pre-loaded nucleo-cassette. Based on the cell counts, the graph of ln (X/X_0_) over time was plotted and the exponential phase was determined visually, as the linear part of the curve. Linear regression was performed on this part of the curve, and the specific growth rate (*μ*) was derived as the slope.

The DT was calculated as before from eq. (2)

### Imaging of FAPs on microcarriers

Homogeneous samples containing medium, microcarriers and cells were stained with Hoechst (dilution 1:626, 33342 392/440, ThermoFisher) and Ethidium homodimer (dilution 1:300, E1169, Invitrogen™) and imaging was performed with the use of an EVOS™ Cell Imaging System (M5000, Invitrogen™).

### FAPs differentiation

Differentiation of FAPs into adipocytes (Figure 1, steps 4–7) was performed as previously described (Dohmen et al., 2022). Briefly, FAPs were suspended in 0.5% high viscosity non-functionalised alginate solution (W201502, Sigma-Aldrich) at 3 × 10^7^ cells·ml^-1^. Cell/alginate suspension was injected into a gelation buffer (66 mM CaCl_2_, 10 mM HEPES). The microfibers were washed with a washing buffer (1 mM CaCl_2_ in DMEM/F12) and they were seeded into 24 well plates at 1 × 10^6^ cells·ml^-1^. The plates were incubated at 37°C and shaken at 70 rpm on an orbital shaker platform. The medium was exchanged at various intervals depending on the experimental condition, by aspirating the liquid from the well until dry and exchanging with fresh medium. Media were sampled for metabolic analysis and stored at −20°C prior to measurement.

### Metabolic analysis

During proliferation and differentiation cultures, medium was sampled before and after medium exchanges and centrifuged for 5 min at 350 G. Metabolite analysis was performed with a bioanalyzer (CEDEX™ Bio, Roche) and quantification of amino acids was performed by quantitative UPLC-MS/MS analysis of underivatized amino acids, as previously described (Waterval et al., 2009).

Specific production/consumption rates of metabolites were calculated for proliferating cells by first graphically determining the exponential phase to determine *μ* (as described above), and applying the following equation:

$$q_{met}=\frac{\mu }{{Χ}_{0}}\times \frac{C_{2}-C_{1}}{e^{\mu t}-1}$$ (3)

q_met_: specific metabolite flux (fmol·cell^-1^·h^-1^), μ: specific growth rate during exponential phase (h^-1^), C_1_: concentration of metabolite at the start of the exponential phase (fmol·ml^-1^), C_2_: concentration of metabolite at the end of the exponential phase, after correcting for the medium exchanges (fmol·ml^-1^), X_0_: cell concentration at the beginning of the exponential phase (cells·ml^-1^), t: duration of exponential phase (h).

For differentiating cells, the following equation was used to calculate the metabolite production/consumption rates:

$$q=\frac{C_{2}-C_{1}}{{t\times Χ}_{0}}$$ (4)

q_met_: specific metabolite flux (fmol·cell^-1^·h^-1^), C_1_: concentration of metabolite before medium exchange (fmol·ml^-1^), C_2_: concentration of metabolite after medium exchange (fmol·ml^-1^), X_0_: seeding cell density (cells·ml^-1^), t: duration of culture (h).

### Imaging of differentiated FAPs

After 28 days, the cell-laden alginate fibres were fixed with paraformaldehyde, stained for nuclei and lipid droplets and imaged with a confocal microscope (TCS SP8, Leica Microsystems), using a 25×/1.00 objective lens and 5 µm Z-steps. Nuclei counts and lipid volumes (LV) were quantified using a custom python script (contact authors for details) for three separate images per sample. To calculate the LV per cell, the total quantified LV was divided by the number of nuclei.

### Ammonium chloride cytotoxicity

Proliferating FAPs were seeded in Gluc/GlnX PM on Cytodex-1 microcarriers in a 24-wells plate set-up, as described above. After 24 h, cells were spiked with 3–7 mM of ammonium chloride (Figure 1, step 2). For toxicity testing during differentiation, cells were seeded in the alginate microfibers as described earlier, in Gal/Pyr DM spiked with 2–6 mM of ammonium chloride (Figure 1, step 7). Immunofluorescence, cell counts, and lipid analysis were performed as described above.

### Statistical analysis

Prism 9.1.0 (GraphPad) was used for statistical analysis. Comparison between two groups was performed using an unpaired *t*-test. Analysis between three or more groups was performed using either one-way ANOVA, two-way ANOVA and mixed ANOVA models, dependent on the specifics and desired comparisons for each experiment. The ANOVA models were combined with Dunnett’s or Šídák’s multiple comparison tests. Pearson correlation was used for correlation analysis. Details on the performed statistics can be found in the figure legends.

## Results and discussion

### Proliferation of FAPs in spinner flasks in control medium (Gluc/GlnX)

**FIGURE 2:**
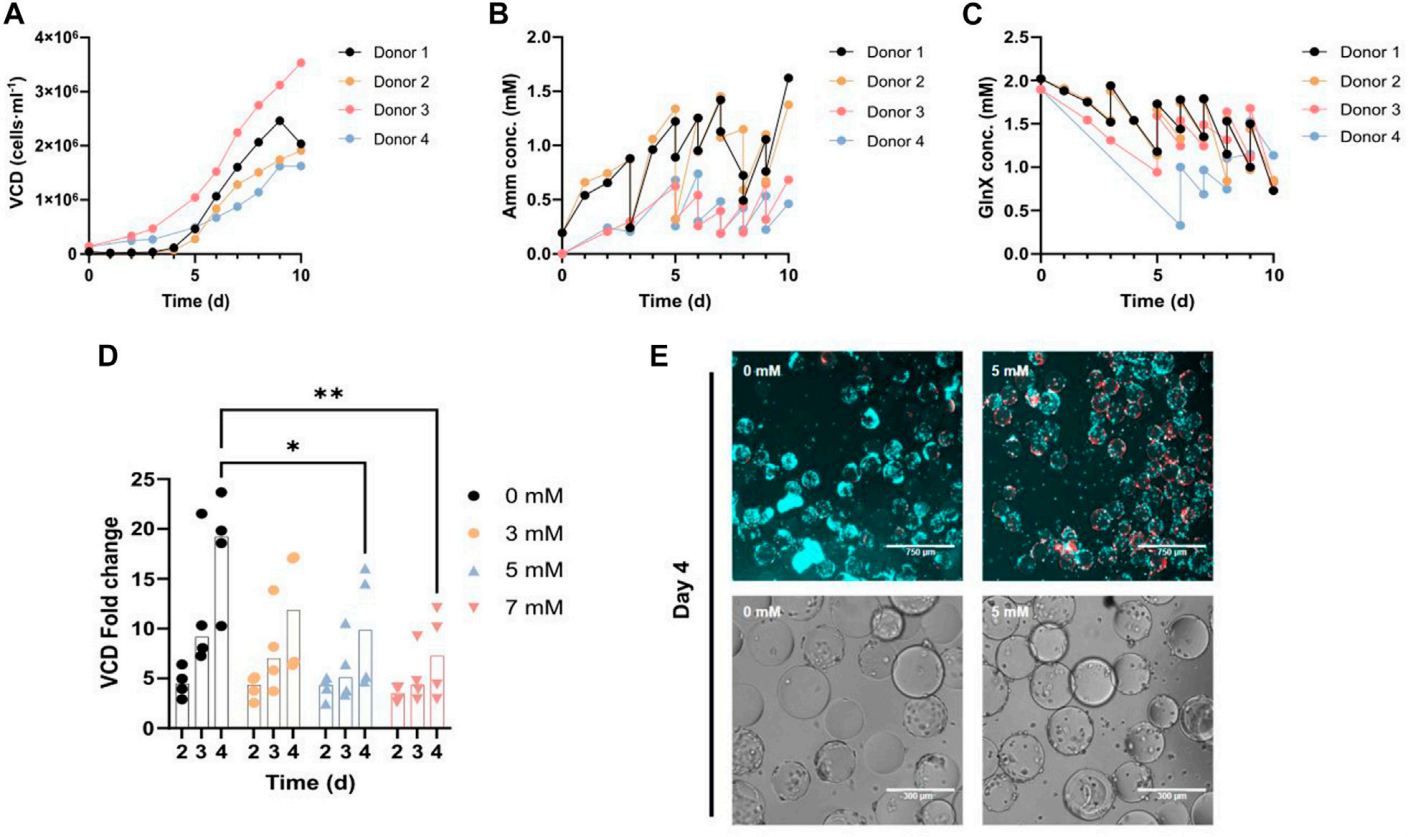
**(A–C)** FAPs in spinner flask culture using control PM for 4 different donors. The data points represent an average of 2-3 technical replicate cell counts or metabolic measurements. **(A)** Growth curve. **(B, C)** Amm and GlnX concentration curves. The double measurements (vertical lines) per time point represent a medium change where two measurements were taken for the same time point, before and after the medium exchange. **(D)** Orbital shaker proliferation of FAPs in PM, spiked with 0–7 mM ammonium chloride. Each observation is the average of 2-3 technical replicates. VCD fold change calculated by dividing each day’s cell density by the cell density on day 0. “*” indicate significance using Dunnett’s multiple comparison test (*p = 0.0292 for 0 mM vs 5 mM on day 4, **p = 0.0027 for 0 mM vs 7 mM on day 4) in an ordinary two-way ANOVA, where time (*p* < 0.0001, F = 13.82) is statistically significant, but concentration (*p* = 0.0072, F = 4.693) and their interaction (*p* = 0.4507, F = 0.9836) are not statistically significant. **(E)** Fluorescence and brightfield microscopy images of FAPs proliferated in PM spiked with ammonium chloride, in the orbital shaker setup; Blue = Hoechst, Red = Ethidium homodimer.

We first investigated Amm metabolism in control PM using Gluc and GlnX in 100 mL spinner flasks. Cells proliferated steadily on Cytodex-1 microcarriers until confluence (Figure 2A). For both donors, the average DT during exponential growth was 25.7 h. Metabolite analysis of the supernatant revealed continuous consumption of GlnX with a concomitant increase of Amm concentration (Figures 2B, C). Through daily medium exchanges, Amm levels were kept below 1.5 mM, as it has been previously reported to be toxic from 2 mM onwards for several cell lines (Hassell et al., 1991). Medium was exchanged up to 10 times (each time replenishing ∼80% of the volume) until a cell density of 2 × 10^6^ cells·ml^-1^ was reached. Applying this feeding regime, GlnX was never depleted in the medium.

### Amm cytotoxicity on proliferating FAPs

To study the effect of increased Amm levels on proliferating and also differentiating FAPs, cells were spiked with increasing levels of ammonium chloride. Proliferating FAPs showed significant growth inhibition from 5 mM onwards on day 4 (Figures 2D, E) in agreement with studies performed on other primary cell types also showing that Amm levels higher than 3 mM are inhibitory to proliferation (Hassell et al., 1991; Schneider, 1996; Schop et al., 2009).

Nevertheless, it has to be noted that toxicity studies of this kind, with a sudden introduction of Amm, do not necessarily directly translate to cell cultures where Amm is secreted by the cells and accumulated at a slower rate. Cells in non-spiked environments might be able to adapt to increasing Amm levels, as described before (Matsumura et al., 1991). Therefore, the critical values found during spiking experiments can only give an indication of Amm toxicity levels for this specific cell type and culture condition.

Based on the cell growth and metabolic data of these spinner flask cultures (Figures 2A, B), an average Amm production rate of q_Amm_ = 56.3 ± 17.3 fmol·cell^-1^·h^-1^ with an average DT of 25.7 ± 1.7 h was calculated. Using this Amm production rate and a hypothetical achievable mid-process cell density of 7 × 10^6^ cells·ml^-1^ (assuming a target end process cell density would be in the range of 10^7^ cells·ml^-1^ (Specht, 2020; Pasitka et al., 2022), we can calculate that a critical concentration of 5 mM of Amm would be reached within 11 h, rendering a medium change or a switch to medium perfusion necessary at this point, to avoid build-up of inhibitory concentrations. Apart from the obvious downsides of frequent medium changes and medium perfusion (i.e., cost, contamination risk), valuable signalling molecules and growth factors produced by the cells would also be removed through that process. Hence, a strategy for reducing Amm build-up was deployed.

### Short-term proliferation on Cytodex-1 in waste reducing medium

**FIGURE 3:**
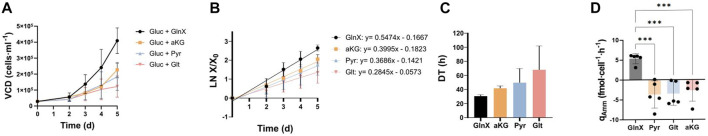
Short-term proliferation of FAPs on Cytodex-1 in the orbital shaker setup in PM with Gluc and GlnX, or with substitutes of the latter, aKG/Pyr/Glt. **(A)** Growth curves. Data points show averages from three donors; each count is a mean of 2-3 technical replicates. Error bars represent standard deviations between three biological replicates. **(B)** Logarithmic growth curves deriving from Figure 3A. Error bars represent standard deviations. A mixed effects model analysis reveals no statistical impact of substrate (*p* = 0.1714; F = 2.155), a significant impact of time (*p* < 0.0001, F = 136,9) and significant effect of their interaction (*p* = 0.0074, F = 2,939). **(C)** Average DTs during proliferation, calculated from the slopes of the regression lines of Figure 3B. Bars show the average DTs from five donors. Error bars represent standard deviation. Data was analysed using an ordinary one-way ANOVA. No statistically significant difference was found between groups (*p* = 0.2100, F = 1.888). **(D)** Specific Amm production rates. Bars show the average from five donors. The individual data points from each donor are displayed. Error bars represent standard deviation. Dunnett’s multiple comparison test reveals significant differences between means: GlnX vs Pyr (***p = 0.002), GlnX vs Glt (***p = 0.003) and GlnX vs aKG (***p = 0.007).

FAPs were screened in a short-term experiment where they were grown for 5 days on Cytodex1 microcarriers, using aKG (10 mM), Pyr (10 mM) and Glt (10 mM) as alternative substrates to GlnX, so as to reduce Amm formation (Figure 3A). Each substrate was initially combined with either high Gluc (17 mM) or low Gluc concentration (5 mM) (Supplementary Figure S2) but since there was no difference observed between the two in terms of both VCD (Supplementary Figures S2A, B) and q_Amm_ (Supplementary Figure S2C), the data for the two Gluc levels were combined.

Pyr and aKG seem to be suitable substrates for FAPs in GlnX-free PM, showing desirable DTs (Figure 3C) while producing no Amm (Figure 3D). The GlnX containing conditions showed a positive Amm production rate of around 5 fmol·cell^-1^·h^-1^ for all three donors, while the GlnX-replacement substrate conditions did not. The specific Amm production rate of the GlnX condition in this orbital shaker experiment was lower than the one calculated previously in the spinner flask culture, possibly due to the different duration of the exponential growth phase and the difference of the setup itself.

Gluc combined with aKG and Pyr led to most satisfactory cell growth during short-term proliferation, with DTs that are on par with and not statistically different from the GlnX containing control and could therefore be suitable for long-term testing and upscaling of cultured meat production.

It is worth noting that in all conditions without GlnX, no or even negative Amm production was observed, indicating that Amm might be taken up by FAPs (Figure 3D) during proliferation. Amm utilisation in Gln deprived environments has been described before (Lie et al., 2019) where it was hypothesised that it could be utilised as an alternative source of nitrogen for Huh7, T47D and MCF7 cells and HEK293. The mechanism of Amm utilisation is possibly through the production of Glt (Takeuchi et al., 2018). This reaction though requires the presence of glutamate dehydrogenase (GDH) and aKG. Briefly, GDH catalyses the reaction of aKG with Amm, generating Glt, which then by further reacting with Amm and catalysed by Gln synthetase, generates Gln (S. Liu et al., 2018; Zhang et al., 2017). GDH is a mitochondrial enzyme found primarily in skeletal muscle (Washington & Van Hoosier, 2012) and it has been reported that the presence of Amm, stimulated growth in breast cancer cells, by means of GDH-catalysed Amm uptake (Supplementary Figure S1) (Spinelli et al., 2017). This recycling mechanism is suggested to support the synthesis of amino acids. However, in the case of primary fibroblast cells, although Amm was also taken up, the cells’ proliferation remained unchanged. Another study found a positive effect of Amm on myotube formation during avian myogenesis (Stern et al., 2019) and here also the authors suggested intracellular Gln synthesis as a possible way of Amm utilisation. In mouse cells however, they found no such effect. Lastly, the administration of 5 mM Amm to primary MSCs led to improved cell proliferation and increased Gln synthetase expression, suggesting that MSCs can utilise Amm to produce Gln (Y. Liu et al., 2022).

Taken together, it seems possible that Amm is taken up and metabolically utilised; yet it is not fully understood which cells have the potential of Amm utilisation and what exactly is the underlying mechanism. It does seem though, that primary FAPs can take up Amm, in the absence of Gln, and also potentially utilise it as a substrate during proliferation.

### Differentiation of FAPs in Gln-based DM using reduced medium volume

After the successful reduction of Amm production during proliferation, we proceeded to test Amm formation during adipogenesis.

**FIGURE 4:**
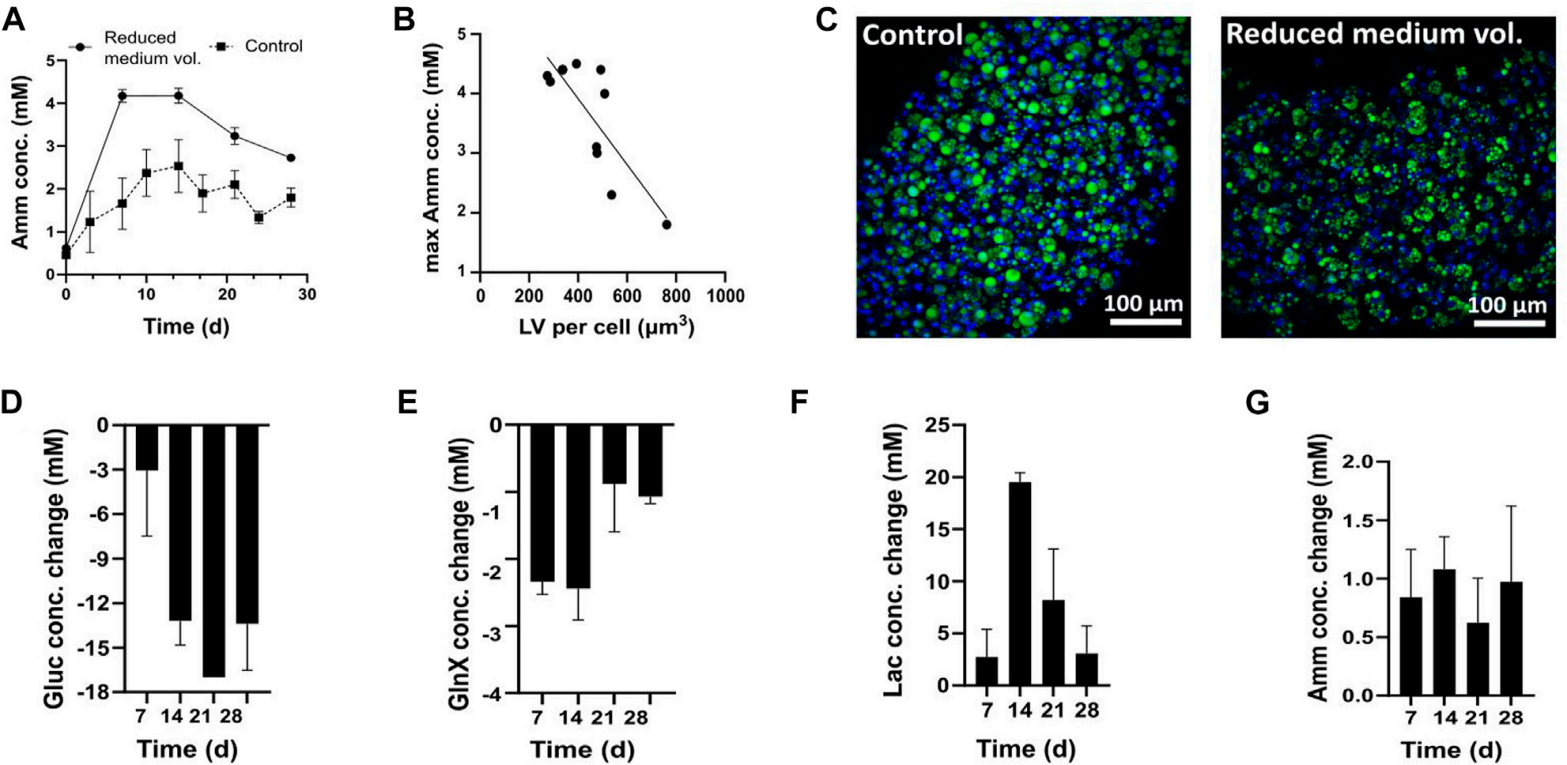
**(A–C)** FAPs differentiated in DM containing 4 mM Gln. **(A)** Amm accumulation in reduced medium and control conditions. Each time point represents the average of 5 technical replicates from one donor and error bars represent the standard deviation. The values presented are the ones before medium change. At each sampling point, a medium exchange was performed, for which the concentration was brought back to the day 0 value, not depicted on the graph. **(B)** Relationship between Amm accumulation and adipogenesis. Pearson’s correlation was calculated to estimate the linear correlation between maximum recorded Amm values on day 14 and maximum recorded LV obtained on day 28 (*R* ^2^ = 0.6562, *p* < 0.0025, n = 10). Data are shown as single Amm and average LV per cell measurements. **(C)** Confocal microscopy images of adipocytes in control and reduced medium volume, day 28; Green = BODIPY, blue = Hoechst. **(D–G)** Gluc, Lac, GlnX and Amm profiles during adipogenic differentiation in DM containing 4 mM GlnX. Bars represent averages from 3 donors and error bars show the standard deviation.

Initially, different medium exchange frequencies were tested; a full medium exchange was performed either every 2–3 days (controls) or every 7 days (reduced medium volume) (Figure 4A). The analysis of Amm levels before every medium exchange revealed that conditions with more frequent medium exchanges did not exceed 3 mM of Amm (Figure 4A), while conditions with less frequent, weekly, medium exchanges reached average concentrations of 4.2 ± 0.1 mM on day 7 and 4.2 ± 0.2 mM on day 14. For both groups, Amm levels reached a plateau by day 14 and less Amm formation was observed thereafter (Figure 4A). This was interpreted as a decreasing dependency of FAPs on nitrogen upon progression of adipogenesis, which was also backed by the corresponding GlnX data (not shown).

Confocal imaging showed smaller lipid droplets in reduced medium volume conditions than in the control conditions (Figure 4C). All microfibers were compared in terms of LV per cell and the negative correlation between lipid droplet size and Amm concentration was found to be statistically significant (*p* < 0.0025) (Figure 4B). We therefore hypothesised that high Amm values in standard DM might be a reason for the decreased lipid droplet formation.

A subsequent experiment was performed using Gln-free DMEM/F12, supplemented with 4 mM GlnX and the fluxes of Gluc, Lac, GlnX and Amm were studied. This time, GlnX was used instead of Gln, which is more stable, and the reduced and therefore more economical, medium exchange frequency of every 7 days was applied. Indeed, it was found that Amm accumulation reached lower maximum values (<2 mM) than when using Gln (Figure 4G).

All cultures started at 17 mM of Gluc. A steady increase of Gluc consumption was observed throughout the culture, peaking on day 21, where Gluc was found to be entirely depleted (Figure 4D).

All cultures started at 0 mM Lac and high Lac formation was observed until day 14 but decreased thereafter (Figure 4F). After day 21, the Lac production no longer mirrored the Gluc consumption. As high Gluc uptake and decreased Lac formation is observed at the same time, we hypothesise that FAPs may switch to Lac consumption once Gluc levels are very low in the medium or that Lac formation is avoided altogether. There are indications that Lac is not just a metabolic waste product but can be used as a carbon source by mammalian cells (Ferguson et al., 2018; Rabinowitz & Enerbäck, 2020). For example, uptake of Lac has been previously reported for muscle (Hamann et al., 2001; Kelley et al., 2002) and CHO cells (Zagari et al., 2013).

Alternatively, FAPs might be switching from glycolysis towards oxidative phosphorylation, which would lead the Pyr produced by glycolysis to enter the TCA cycle for further oxidation, thus avoiding Lac production. Previous research shows that adipogenic differentiation is associated with increased mitochondrial oxidative activity (Luo et al., 2007; Ryu et al., 2013). Likewise, Hofmann et al. report an increase in mitochondrial biogenesis related genes involved in oxidative metabolism for differentiating hMSCs (Hofmann et al., 2012), which supports our suggestion that FAPs could be switching their metabolism towards oxidative phosphorylation. The mechanism underlying the Lac generation and uptake observations can be further investigated using for instance radiolabelled Lac (Y. Wang et al., 2020).

Lastly, regarding GlnX and Amm, all cultures started at 4 mM and 0 mM respectively. Differentiating FAPs showed an increased GlnX uptake for the first 2 weeks (∼2 mM per week), which decreased thereafter (∼1 mM per week) until day 28 (Figure 4E). Amm levels throughout the culture were stable, with maximum values before each medium change fluctuating around 1 mM (Figure 4G). These findings align with the previously observed reduced Amm production after 14 days of culture in 4 mM Gln (Figure 4A) and strengthen the hypothesis that FAPs may, at some point during the culture, no longer rely on Gln (or GlnX for that matter) as much as they do in the beginning of the differentiation process but might instead use Gluc and even Lac as a substrate as adipocytes mature.

### Long-term passaging of FAPs and differentiation in Amm reducing media

To study the long-term effect of Amm reducing strategies, an experimental design was set up with aKG, Glt, Pyr and Gal substrates in a multipassage FAPs proliferation and differentiation setup, comparing GlnX-containing and GlnX-free based PM and DM. FAPs were first cultured in PM containing combinations of Gluc with GlnX (control) and GlnX replacements (aKG, Pyr and Glt) over 5 passages using a planar system. After passages 5 and 7, cells were seeded for adipogenic differentiation in either control medium containing Gluc/GlnX, or in non-ammoniagenic media containing Gal/Pyr and Gluc/Pyr. The combinations Gal/Pyr and Gluc/Pyr were chosen for differentiation because previously, several combinations of Gluc or Gal combined with either Pyr or Glt had been tested and these ones showed to favour adipogenesis (Supplementary Figure S3A).

**FIGURE 5:**
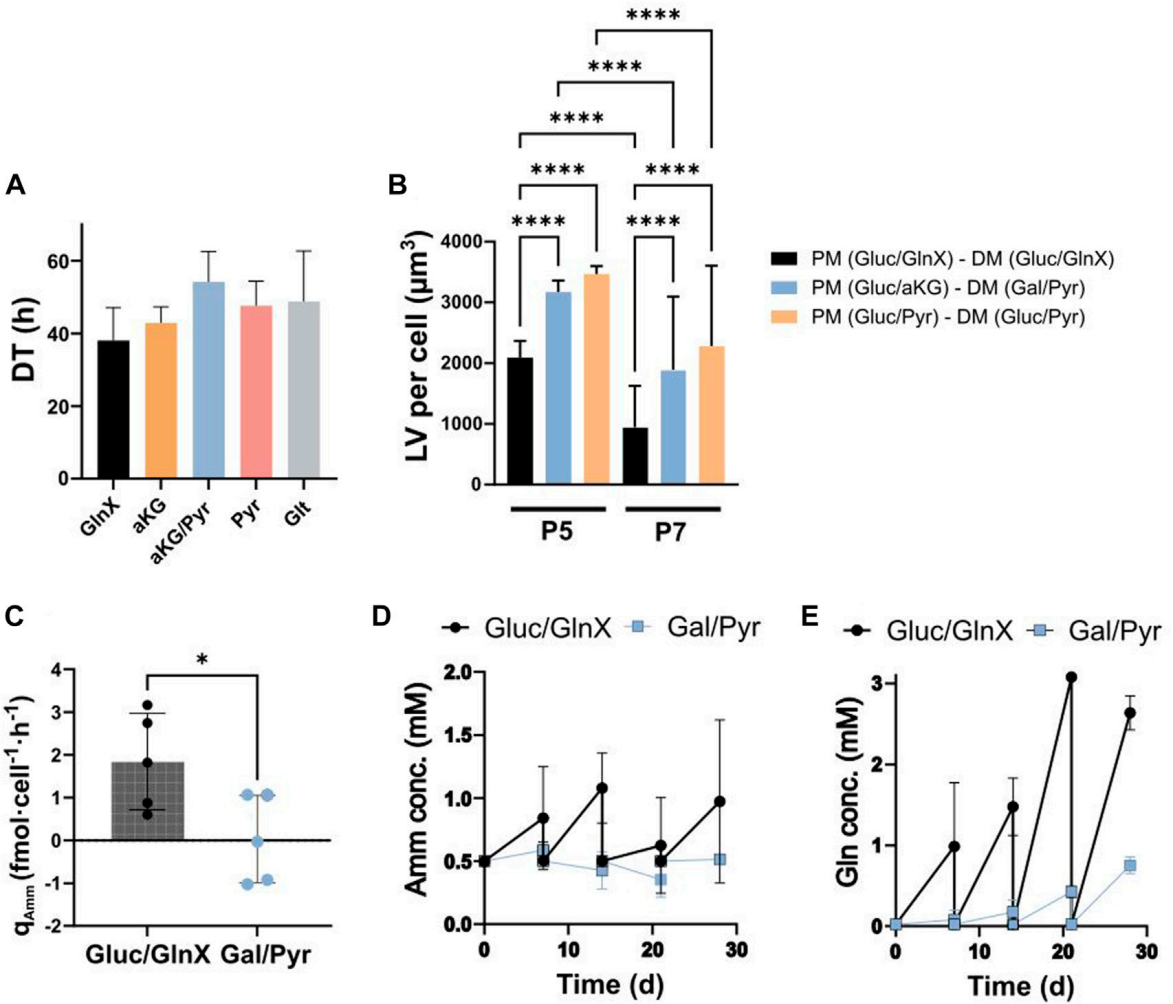
**(A)** Average DTs during long-term passaging of FAPs in GlnX, aKG, aKG/Pyr, Pyr and Glt supplemented PM. Average of cell counts over 5 passages of two donors were used for the calculation of the DTs. Data is represented as the mean of 5 observations per condition. Error bars represent standard deviation. Data was analysed using a two-way ANOVA, indicating no significant effect of substrate (*p* = 0.1704), passage number (*p* = 0.1913) and their interaction (*p* = 0.8635). Dunnett’s multiple comparison test indicates no significant difference between column means. **(B)** Differentiation quality in LV per cell. Cells were proliferated and differentiated (after passage 5 and 7) in ammoniagenic and non-ammoniagenic media. For fat quantification, 25 independent images were taken per condition and per donor, deriving from 2 donors; **** indicate statistical significance using Šídák’s multiple comparison test between conditions column means (*p* < 0.0001, n = 50). Two-way ANOVA reveals significant effects of d onor (*p* < 0.0001, F = 74.29), substrate (*p* < 0.0001, F = 83.15) and their interaction (*p* < 0.0001, F = 22.81). The individual donor values can be found in Supplementary Figure S4B. **(C)** q_Amm_ in Gluc/GlnX vs Gal/Pyr DM. Bars represent average rates of 5 donors. Error bars represent standard deviation. * indicates statistically significant difference between means of 5 donors using an unpaired *t*-test (*p* < 0.0283, *n* = 5). **(D, E)** Amm and Gln concentration curves during 28 days of differentiation in Gluc/GlnX vs Gal/Pyr differentiation media; datapoints represent the averaged concentration in medium samples from 3 donors. Error bars represent standard deviation.

To evaluate the long-term performance of each substrate, the cell counts from five passages were averaged for each substrate. All GlnX replacements succeeded to maintain growth of FAPs for over 5 passages (Figure 5A). FAPs proliferated with an average DT of 38.0 ± 9.1 h (*n* = 5) in GlnX-medium. The alternative substrates led to similar average DTs and are ranked from lowest to highest: aKG with 42.9 ± 4.4 h, Pyr with 47.6 ± 6.8 h, Glt with 48.8 ± 13.9 h and aKG/Pyr with 54.2 ± 8.4 h (n = 5 for all). No significant difference in growth rates was observed between the tested substrates and the control.

After proliferation, cells were seeded for differentiation. Since we tested two donors in 4 proliferation substrates and subsequently split each proliferation culture to 2-3 DM formulations, at two proliferation stages (P5 and P7), we obtained 36 observations from which we only show the most relevant ones (Figure 5B). The full data set can be found in the Supplementary Material (Supplementary Table S2; Supplementary Figures S4A).

It was observed that FAPs expanded in Gluc/aKG and differentiated in Gal/Pyr medium, as well as cells expanded in Gluc/Pyr and differentiated in Gluc/Pyr medium, all non-ammoniagenic media, showed a significant increase in lipid accumulation compared to the control, up to 2.1 and 2.3-fold respectively (Figure 5B). This result was consistent between P5 and P7 cells, albeit P7 cells in general showed a significantly decreased differentiation capacity in all medium formulations, probably due to cell age (Supplementary Figure S4A).

These data support the earlier findings on Gln utilization during adipogenesis (Figure 4) and suggest that Gln is not beneficial to FAPs adipogenic differentiation. Existing reports on the relationship between Gln and adipogenesis are conflicting. It was previously found that 10 mM of Gln reduced lipid content, pro-inflammatory protein and gene responses in adipocytes and white adipose tissue expansion (Petrus et al., 2020). Yu et al. also reported that neither Gln consumption nor Gln synthetase activity were altered during adipogenesis of skeletal stem cells and that Gln withdrawal increased adipogenic marker gene expression and lipid accumulation during differentiation (Yu et al., 2019). In contrast, Velickovic et al. found that Gln is an essential energy source during adipogenic differentiation and lipid droplet formation of mouse MSCs, as it was found beneficial in a dose dependent manner whereas differentiation was suppressed upon Gln deprivation, without any Gln replacements present (Velickovic et al., 2020). In 3T3-L1 adipocytes, it was found that Gln was produced and secreted in dependency on Gln synthetase, with its inhibition leading to impaired differentiation and increased lipolysis. Inhibition of Gln synthetase was rescued by exogenous Gln and it was therefore concluded to be a necessary compound for differentiation and a regulator for lipolysis (Okuro et al., 2021).

From these contradictory findings we get the impression that the effect of Gln on adipogenesis might perhaps be cell-type, setup, context, concentration and medium formulation dependent.

We observed that combinations of Gal, Pyr and aKG act as supporting agents for FAPs adipogenesis. The effect of Pyr has previously been described in a study using fibroblasts, where it prevented mitochondrial dysfunction and senescence (Kim et al., 2018). Another study reported, in alignment with our findings, that Pyr increased lipid droplet formation in adipocyte-like 3T3-L1 cells (Hwang et al., 2016).

Additionally, aKG may also act as an antioxidant and an Amm scavenger during proliferation of mammalian cells (S. Liu et al., 2018), therefore positively affecting cell state and subsequent differentiation.

Here, a positive effect of Gal on adipogenesis was observed. When FAPs were differentiated in Gal/Pyr, adipogenesis was enhanced, as opposed to when Pyr was used alone, where no differentiation was observed (Supplementary Figure S5A). Since in our experiments Gal was not found to be consumed (Supplementary Figure S5B) we hypothesise that it might have a signalling effect towards adipogenesis or an anti-apoptotic effect.

There are very few reports that suggest a positive effect of Gal on adipogenesis. One study from 1974, found that Gal enhanced Gluc uptake of rat adipocytes, possibly because of a shift in the red-ox state of the cells (Naito & Okada, 1974).

Another study on hepatocytes reported that administration of Gal prevented TNF-α induced apoptosis, by inducing nuclear factor kappa B (NF-κB) signalling pathways (Y. Liu et al., 2015), which for adipocytes has been described to regulate adipocyte function and differentiation (Weidemann et al., 2016). Furthermore, TNF-α is known to decrease free fatty acid uptake into the cell and synthesis of triglycerides, while at the same time increasing lipolysis (Sethi & Hotamisligil, 1999; Weidemann et al., 2016). It is therefore possible that Gal plays a supporting role in FAPs adipogenesis and free fatty acid uptake. Conversely to our findings, a previous report showed that Gal did not promote adipogenic differentiation in a mouse cell line (Krishna et al., 2020). Also here, the effect of Gal on cells might be cell-type and/or medium formulation dependent.

Supernatant analysis showed that Amm production in Gal/Pyr medium was significantly decreased compared to Gluc/GlnX control DM (Figure 5C). This is advantageous, as it provides the possibility to extend medium usage by allowing bolus feeding of depleted substrates. This would allow the extended exposure of cells to their own secreted proteins and cytokines, which has been shown to stimulate adipose tissue metabolism and lipid accumulation, through induction of inflammatory stimuli, autocrine and paracrine effects (Coppack, 2001).

During 28 days of culture, FAPs accumulated Amm in the Gluc/GlnX medium, while in Gal/Pyr medium, Amm concentrations marginally increased until day 7 and subsequently decreased until day 21 (Figure 5D). It has to be noted that Amm concentrations were measured at 0.5 mM already from day 0, in both Gln-free and Gln containing DM. A possible reason for the presence of ammonia in the medium could be through amino acid degradation in the commercial basal. Overall, the Amm production rates during adipogenic differentiation significantly decreased compared to the GlnX-containing control medium (Figure 5C).

Whereas Gln build up in the Gluc/GlnX medium was an expected result, as it is a consequence of GlnX being cleaved into Gln and alanine by the cells, surprisingly, Gln was also slightly building up in the Gal/Pyr medium (Figure 5E), suggesting production of Gln by the cells. Amino acid analysis of the supernatant of the Gal/Pyr based FAPs differentiation culture, revealed that asparagine, proline and most severely aspartic acid were rapidly consumed (Supplementary Figures S3B–D). Aspartate aminotransferase, a transaminase enzyme, is involved in the conversion of aKG and aspartate to oxaloacetate and Glt (Washington & Van Hoosier, 2012), from which Gln could subsequently be synthesised. Another possible mechanism for Gln production could be via GDH as described earlier in the short-term proliferation study (Figure 3D). These processes would therefore explain the detection of Gln and Glt in the medium, whilst these were not added in the formulation. Looking at both Amm uptake and Gln production, it could be that FAPs recycle Amm to produce Gln. The combined presence of Gluc and Gln has previously been described to be essential for cell growth and survival, as one is thought to directly depend on the other. For several cell lines it has been found that the CtBP-SIRT4-GDH axis, a pathway that mediates the glycolysis’ impact on Gln metabolism, is a mechanism for cell survival (L. Wang et al., 2018). It has also been observed that the presence of Gln dictates Gluc uptake and mitochondrial anaplerosis of pancreatic cancer cells (Kaadige et al., 2009).

The current study challenges these hypotheses, by successfully replacing both Gln and Gluc during adipogenic differentiation of FAPs.

### Cytotoxicity of Amm on differentiating FAPs

To test the sensitivity of differentiating FAPs to ammonium chloride in a low Amm background, cells were induced for adipogenesis in non-ammoniagenic Gal/Pyr medium and spiked with 2–6 mM of ammonium chloride.

**FIGURE 6:**
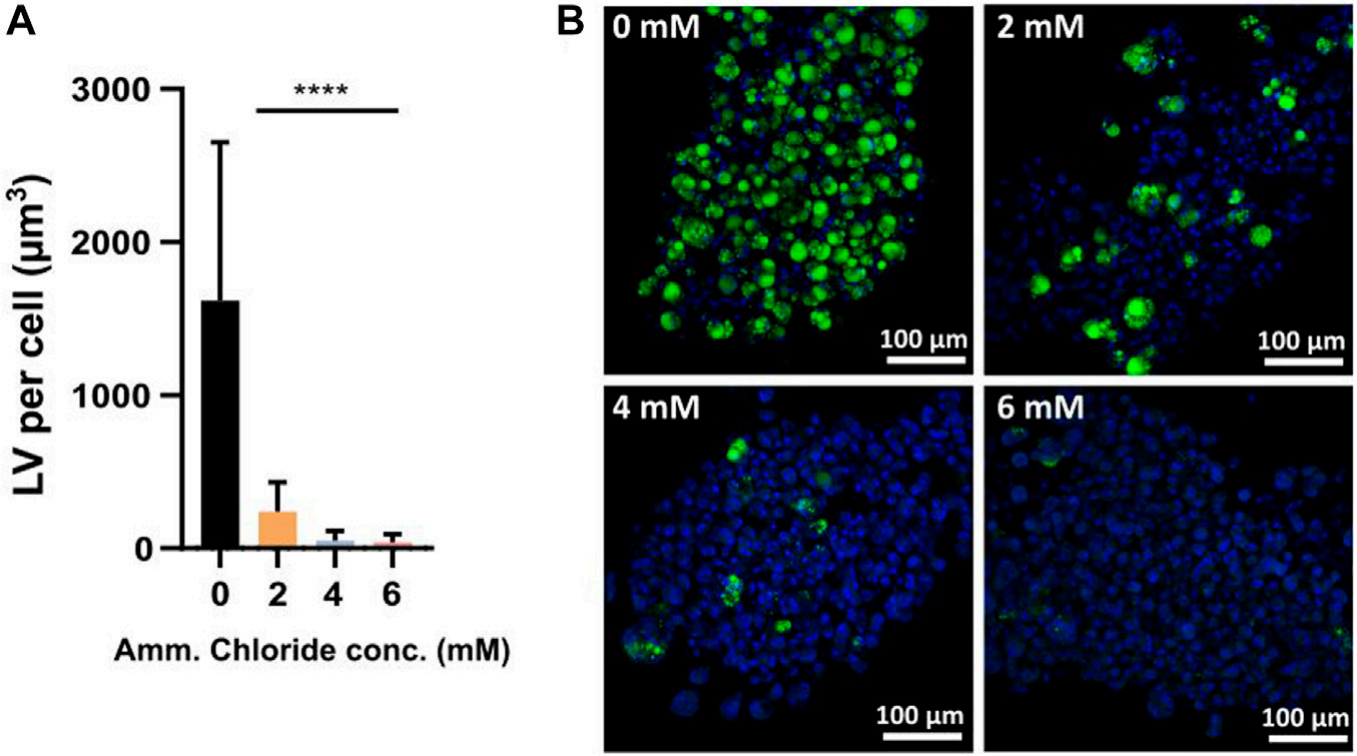
**(A)** LV per cell for samples spiked with 0–6 mM of ammonium chloride. Bars represent the average from 3 donors (means of 5 independent images each). Error bars represent the standard deviation. Statistically significant difference of 2–6 mM ammonium chloride when compared to the 0 mM control by two-way ANOVA (*p* < 0.0001, n = 3); donor (*p* < 0.0001, F = 13.91), treatment (*p* < 0.0001, F = 28.4) and donor × treatment interaction (*p* < 0.0001, F = 108.1) are all statistically significant. Dunnett’s multiple comparison test reveals significant differences between control mean (0 mM) and all other means (2–6 mM) (all *p* < 0.0001) **(B)** Confocal microscopy images of FAPs adipogenic differentiation in Gal/Pyr DM spiked with 0–6 mM of ammonium chloride; Green = BODIPY, blue = Hoechst.

Confocal fluorescent imaging of differentiated FAPs analysis showed significantly reduced adipogenesis, expressed as LV per cell (μm^3^), from 2 mM ammonium chloride onwards (Figures 6A,B). This data is aligned with the previously acquired data in Gln containing medium (Figures 4A,B) showing a negative correlation between increasing Amm levels and decreased LV, confirming that it is indeed the presence of Amm, which is inhibiting the differentiation. Earlier we showed that Amm (≥5 mM) is inhibitory to proliferation (Figure 2D), and we confirm the same applies for Amm (≥2 mM) during adipogenic differentiation. This is the first study, to our knowledge, demonstrating an inhibitory effect of Amm on *in vitro* adipogenesis.

## Conclusion

Once reaching critical concentrations, Amm significantly decreases growth rates and differentiation quality of primary FAPs. Hence, to achieve high cell density cultures, it is necessary to reduce its accumulation. Replacing Gln and GlnX with non-ammoniagenic compounds such as aKG, Pyr and Glt allows for comparable proliferation rates of FAPs over several passages, while also maintaining their differentiation capacity. Furthermore, non-ammoniagenic DM containing Gal/Pyr as alternative substrates to Gluc and Gln, significantly improves FAPs’ adipogenic differentiation. Overall, the adipogenic capacity was improved by 2.1-fold when cells were proliferated and differentiated in non-ammoniagenic media compared to glutamine containing media. The beneficial effect of the non-ammoniagenic media was more pronounced in older, P7 cells (up to 2.3-fold increase) when compared to P5 cells, where a maximum of 1.7-fold increase in fat volume per cell was observed.

The replacement of GlnX led to significantly decreased Amm production rates, in some cases even resulting in Amm uptake, suggesting that primary FAPs not only do not require Gln as an essential nutrient, but on the contrary, they possess all the necessary metabolic pathways to proliferate and subsequently differentiate in a Gln-free medium allowing for decreased Amm accumulation and *de novo* Gln synthesis.

A bioprocess utilising non-ammoniagenic media covering both expansion and differentiation phases, is extremely beneficial during upscaling, as apart from the increased productivity (in terms of fat volume per cell), it also allows for more efficient use of culture media, increasing its life span and reducing the waste of valuable nutrients that takes place as part of the medium exchanges that are performed to prevent Amm induced growth stagnation and inhibition of differentiation. In this way, it contributes to lower cost of goods and improved sustainability.

## Data Availability

The original contributions presented in the study are included in the article/Supplementary Material, further inquiries can be directed to the corresponding author.

## Abbreviations

aKG, alpha-ketoglutarate; Amm, ammonia; DM, differentiation medium; FAPs, fibro-adipogenic progenitor cells; Gal, galactose; GDH, glutamate dehydrogenase; Gln, glutamine; GlnX, GlutaMax; Glt, glutamate; Gluc, glucose; Lac, lactate; LV, lipid volume; PM, proliferation medium; Pyr, pyruvate; VCD, viable cell density.

## References

[B1] Bhat Z. F. Morton J. D. Mason S. L. Bekhit A. E.-D. A. Bhat H. F. (2019). Technological, regulatory, and ethical aspects of *in vitro* meat: A future slaughter-free harvest. Compr. Rev. Food Sci. Food Saf. 18 (4), 1192–1208. 10.1111/1541-4337.12473 33336995

[B2] Braissant O. McLin V. A. Cudalbu C. (2013). Ammonia toxicity to the brain. J. Inherit. Metabolic Dis. 36 (4), 595–612. 10.1007/s10545-012-9546-2 23109059

[B3] Christie A. Butler M. (1999). The adaptation of BHK cells to a non-ammoniagenic glutamate-based culture medium. Biotechnol. Bioeng. 64 (3), 298–309. 10.1002/(sici)1097-0290(19990805)64:3<298::aid-bit6>3.0.co;2-u 10397867

[B4] Coppack S. W. (2001). Pro-inflammatory cytokines and adipose tissue. Proc. Nutr. Soc. 60 (3), 349–356. 10.1079/PNS2001110 11681809

[B5] Corbet C. (2018). Stem cell metabolism in cancer and healthy tissues: Pyruvate in the limelight. Front. Pharmacol. 8, 958. 10.3389/fphar.2017.00958 29403375 PMC5777397

[B6] Demir A. Gunay A. Debik E. (2002). Ammonium removal from aqueous solution by ion-exchange using packed bed natural zeolite. Water SA 28 (3), 329–336. 10.4314/wsa.v28i3.4903

[B7] Ding Y. Sartaj M. (2016). Optimization of ammonia removal by ion-exchange resin using response surface methodology. Int. J. Environ. Sci. Technol. 13 (4), 985–994. 10.1007/s13762-016-0939-x

[B8] Dohmen R. G. J. Hubalek S. Melke J. Messmer T. Cantoni F. Mei A. (2022). Muscle-derived fibro-adipogenic progenitor cells for production of cultured bovine adipose tissue. Npj Sci. Food 6 (1), 6. 10.1038/s41538-021-00122-2 35075125 PMC8786866

[B9] Ferguson B. S. Rogatzki M. J. Goodwin M. L. Kane D. A. Rightmire Z. Gladden L. B. (2018). Lactate metabolism: Historical context, prior misinterpretations, and current understanding. Eur. J. Appl. Physiology 118 (4), 691–728. 10.1007/s00421-017-3795-6 29322250

[B10] Gambhir A. Korke R. Lee J. Fu P.-C. Europa A. Hu W.-S. (2003). Analysis of cellular metabolism of hybridoma cells at distinct physiological states. J. Biosci. Bioeng. 95 (4), 317–327. 10.1016/S1389-1723(03)80062-2 16233414

[B11] Genzel Y. Ritter J. B. König S. Alt R. Reichl U. (2008). Substitution of glutamine by pyruvate to reduce ammonia formation and growth inhibition of mammalian cells. Biotechnol. Prog. 21 (1), 58–69. 10.1021/bp049827d 15903241

[B12] Ha T. K. Lee G. M. (2014). Effect of glutamine substitution by TCA cycle intermediates on the production and sialylation of Fc-fusion protein in Chinese hamster ovary cell culture. J. Biotechnol. 180, 23–29. 10.1016/j.jbiotec.2014.04.002 24721212

[B13] Hamann J. J. Kelley K. M. Gladden L. B. (2001). Effect of epinephrine on net lactate uptake by contracting skeletal muscle. J. Appl. Physiology 91 (6), 2635–2641. 10.1152/jappl.2001.91.6.2635 11717229

[B14] Hanga M. P. de la Raga F. A. Moutsatsou P. Hewitt C. J. Nienow A. W. Wall I. (2021). Scale-up of an intensified bioprocess for the expansion of bovine adipose-derived stem cells (bASCs) in stirred tank bioreactors. Biotechnol. Bioeng. 118 (8), 3175–3186. 10.1002/bit.27842 34076888

[B15] Haraguchi Y. Shimizu T. (2021a). Microalgal culture in animal cell waste medium for sustainable ‘cultured food’ production. Archives Microbiol. 203 (9), 5525–5532. 10.1007/s00203-021-02509-x 34426852

[B16] Haraguchi Y. Shimizu T. (2021b). Three-dimensional tissue fabrication system by co-culture of microalgae and animal cells for production of thicker and healthy cultured food. Biotechnol. Lett. 43 (6), 1117–1129. 10.1007/s10529-021-03106-0 33689062

[B17] Hassell T. Butler M. (1990). Adaptation to non-ammoniagenic medium and selective substrate feeding lead to enhanced yields in animal cell cultures. J. Cell Sci. 96 (3), 501–508. 10.1242/jcs.96.3.501 1977752

[B18] Hassell T. Gleave S. Butler M. (1991). Growth inhibition in animal cell culture: The effect of lactate and ammonia. Appl. Biochem. Biotechnol. 30 (1), 29–41. 10.1007/BF02922022 1952924

[B19] Hofmann A. D. Beyer M. Krause-Buchholz U. Wobus M. Bornhäuser M. Rödel G. (2012). OXPHOS supercomplexes as a hallmark of the mitochondrial phenotype of adipogenic differentiated human MSCs. PLoS ONE 7 (4), e35160. 10.1371/journal.pone.0035160 22523573 PMC3327658

[B20] Hubalek S. Post M. J. Moutsatsou P. (2022). Towards resource-efficient and cost-efficient cultured meat. Curr. Opin. Food Sci. 47, 100885. 10.1016/j.cofs.2022.100885

[B21] Hwang J.-S. Kim S.-Y. Jung E.-H. Kwon M.-Y. Kim K.-H. Cho H. (2016). Exogenous sodium pyruvate stimulates adipogenesis of 3T3-L1 cells. J. Cell. Biochem. 117 (1), 39–48. 10.1002/jcb.25244 26053972

[B22] Kaadige M. R. Looper R. E. Kamalanaadhan S. Ayer D. E. (2009). Glutamine-dependent anaplerosis dictates glucose uptake and cell growth by regulating MondoA transcriptional activity. Proc. Natl. Acad. Sci. 106 (35), 14878–14883. 10.1073/pnas.0901221106 19706488 PMC2736411

[B23] Kelley K. M. Hamann J. J. Navarre C. Gladden L. B. (2002). Lactate metabolism in resting and contracting canine skeletal muscle with elevated lactate concentration. J. Appl. Physiology 93 (3), 865–872. 10.1152/japplphysiol.01119.2001 12183479

[B24] Kim J. Y. Lee S. H. Bae I.-H. Shin D. W. Min D. Ham M. (2018). Pyruvate protects against cellular senescence through the control of mitochondrial and lysosomal function in dermal fibroblasts. J. Investigative Dermatology 138 (12), 2522–2530. 10.1016/j.jid.2018.05.033 29959907

[B25] Kolkmann A. M. Van Essen A. Post M. J. Moutsatsou P. (2022). Development of a chemically defined medium for *in vitro* expansion of primary bovine satellite cells. Front. Bioeng. Biotechnol. 10, 895289. 10.3389/fbioe.2022.895289 35992337 PMC9385969

[B26] Krishna M. S. Revathy V. M. Jaleel A. (2020). Adipocytes utilize sucrose as an energy source—effect of different carbohydrates on adipocyte differentiation. J. Cell. Physiology 235 (2), 891–899. 10.1002/jcp.29003 31240708

[B27] Lie S. Wang T. Forbes B. Proud C. G. Petersen J. (2019). The ability to utilise ammonia as nitrogen source is cell type specific and intricately linked to GDH, AMPK and mTORC1. Sci. Rep. 9 (1), 1461. 10.1038/s41598-018-37509-3 30728400 PMC6365639

[B28] Lim Y. Wong N. S. C. Lee Y. Y. Ku S. C. Y. Wong D. C. F. Yap M. G. S. (2010). Engineering mammalian cells in bioprocessing—current achievements and future perspectives. Biotechnol. Appl. Biochem. 55 (4), 175–189. 10.1042/BA20090363 20392202

[B29] Liu S. He L. Yao K. (2018). The antioxidative function of alpha-ketoglutarate and its applications. BioMed Res. Int. 2018, 1–6. 10.1155/2018/3408467 PMC5884300 29750149

[B30] Liu Y. Zhang X. Wang W. Liu T. Ren J. Chen S. (2022). Ammonia promotes the proliferation of bone marrow-derived mesenchymal stem cells by regulating the Akt/mTOR/S6k pathway. Bone Res. 10 (1), 57. 10.1038/s41413-022-00215-y 36028500 PMC9418171

[B31] Liu Y. Zhu L. Liang S. Yao S. Li R. Liu S. (2015). Galactose protects hepatocytes against TNF-α-induced apoptosis by promoting activation of the NF-κB signaling pathway in acute liver failure. Lab. Investig. 95 (5), 504–514. 10.1038/labinvest.2015.34 25751739

[B32] Luo G.-F. Yu T.-Y. Wen X.-H. Li Y. Yang G.-S. (2007). Alteration of mitochondrial oxidative capacity during porcine preadipocyte differentiation and in response to leptin. Mol. Cell. Biochem. 307 (1–2), 83–91. 10.1007/s11010-007-9587-2 17909948

[B33] Martinelle K. Häggström L. (1993). Mechanisms of ammonia and ammonium ion toxicity in animal cells: Transport across cell membranes. J. Biotechnol. 30 (3), 339–350. 10.1016/0168-1656(93)90148-G 7764110

[B34] Matsumura M. Shimoda M. Arii T. Kataoka H. (1991). Adaptation of hybridoma cells to higher ammonia concentration. Cytotechnology 7 (2), 103–112. 10.1007/BF00350916 1367906

[B35] McDermott R. H. Butler M. (1993). Uptake of glutamate, not glutamine synthetase, regulates adaptation of mammalian cells to glutamine-free medium. J. Cell Sci. 104 (1), 51–58. 10.1242/jcs.104.1.51 8095504

[B36] Mehta F. Theunissen R. Post M. J. (2019). Adipogenesis from bovine precursors. Methods Mol. Biol. Clift. N.J.) 1889, 111–125. 10.1007/978-1-4939-8897-6_8 30367412

[B37] Messmer T. Klevernic I. Furquim C. Ovchinnikova E. Dogan A. Cruz H. (2022). A serum-free media formulation for cultured meat production supports bovine satellite cell differentiation in the absence of serum starvation. Nat. Food 3, 74–85. 10.1038/s43016-021-00419-1 37118488

[B38] Naito C. Okada K. (1974). Stimulation of glucose metabolism by galactose in isolated adipocytes from rats. J. Biol. Chem. 249 (6), 1657–1660. 10.1016/s0021-9258(19)42839-1 4856362

[B39] Okuro K. Fukuhara A. Minemura T. Hayakawa T. Nishitani S. Okuno Y. (2021). Glutamine deficiency induces lipolysis in adipocytes. Biochem. Biophysical Res. Commun. 585, 155–161. 10.1016/j.bbrc.2021.11.043 34801935

[B40] Pasitka L. Cohen M. Ehrlich A. Gildor B. Reuveni E. Ayyash M. (2022). Spontaneous immortalization of chicken fibroblasts generates stable, high-yield cell lines for serum-free production of cultured meat. Nat. Food 4, 35–50. 10.1038/s43016-022-00658-w 37118574

[B41] Petrus P. Lecoutre S. Dollet L. Wiel C. Sulen A. Gao H. (2020). Glutamine links obesity to inflammation in human white adipose tissue. Cell Metab. 31 (2), 375–390.e11. 10.1016/j.cmet.2019.11.019 31866443

[B42] Post M. J. Levenberg S. Kaplan D. L. Genovese N. Fu J. Bryant C. J. (2020). Scientific, sustainability and regulatory challenges of cultured meat. Nat. Food 1 (7), 403–415. 10.1038/s43016-020-0112-z

[B43] Rabinowitz J. D. Enerbäck S. (2020). Lactate: The ugly duckling of energy metabolism. Nat. Metab. 2 (7), 566–571. 10.1038/s42255-020-0243-4 32694798 PMC7983055

[B44] Ryu M. J. Kim S. J. Choi M. J. Kim Y. K. Lee M. H. Lee S. E. (2013). Mitochondrial oxidative phosphorylation reserve is required for hormone- and PPARγ agonist-induced adipogenesis. Mol. Cells 35 (2), 134–141. 10.1007/s10059-012-2257-1 23456335 PMC3887907

[B45] Schneider M. (1996). The importance of ammonia in mammalian cell culture. J. Biotechnol. 46 (3), 161–185. 10.1016/0168-1656(95)00196-4 8672289

[B46] Schop D. Janssen F. W. van Rijn L. D. S. Fernandes H. Bloem R. M. de Bruijn J. D. (2009). Growth, metabolism, and growth inhibitors of mesenchymal stem cells. Tissue Eng. Part A 15 (8), 1877–1886. 10.1089/ten.tea.2008.0345 19196147

[B47] Sethi J. K. Hotamisligil G. S. (1999). The role of TNFα in adipocyte metabolism. Seminars Cell and Dev. Biol. 10 (1), 19–29. 10.1006/scdb.1998.0273 10355025

[B48] Sikdar S. K. Sawant S. B. (1994). Ammonia removal from mammalian cell culture medium by ion-exchange membranes. Sep. Sci. Technol. 29 (12), 1579–1591. 10.1080/01496399408007375

[B49] Specht L. (2020). An analysis of culture medium costs and production volumes for cultivated meat. Washington, DC: The Good Food Institute.

[B50] Spinelli J. B. Yoon H. Ringel A. E. Jeanfavre S. Clish C. B. Haigis M. C. (2017). Metabolic recycling of ammonia via glutamate dehydrogenase supports breast cancer biomass. Science 358 (6365), 941–946. 10.1126/science.aam9305 29025995 PMC5748897

[B51] Stern R. A. Dasarathy S. Mozdziak P. E. (2019). Ammonia induces a myostatin-mediated atrophy in mammalian myotubes, but induces hypertrophy in avian myotubes. Front. Sustain. Food Syst. 3, 115. 10.3389/fsufs.2019.00115

[B52] Stout A. J. Mirliani A. B. White E. C. Yuen J. S. K. Kaplan D. L. (2021). Simple and effective serum-free medium for sustained expansion of bovine satellite cells for cell cultured meat. Bioengineering 2021, 446057. 10.1101/2021.05.28.446057 PMC9163123 35654948

[B53] Takeuchi Y. Nakayama Y. Fukusaki E. Irino Y. (2018). Glutamate production from ammonia via glutamate dehydrogenase 2 activity supports cancer cell proliferation under glutamine depletion. Biochem. Biophysical Res. Commun. 495 (1), 761–767. 10.1016/j.bbrc.2017.11.088 29146184

[B54] Tareq K. Miah A. G. Salma U. Yoshida M. Tsujii H. (2007). Effect of amino acids and dipeptides on accumulation of ammonia in the medium during *in vitro* maturation and fertilization of porcine oocytes. Reproductive Med. Biol. 6 (3), 165–170. 10.1111/j.1447-0578.2007.00180.x PMC5904632 29699273

[B55] Velickovic K. Lugo Leija H. A. Surrati A. Kim D.-H. Sacks H. Symonds M. E. (2020). Targeting glutamine synthesis inhibits stem cell adipogenesis *in vitro* . Cell. Physiology Biochem. 54 (5), 917–927. 10.33594/000000278 32946687

[B56] Vergara M. Torres M. Müller A. Avello V. Acevedo C. Berrios J. (2018). High glucose and low specific cell growth but not mild hypothermia improve specific r-protein productivity in chemostat culture of CHO cells. PloS One 13 (8), e0202098. 10.1371/journal.pone.0202098 30114204 PMC6095543

[B57] Vis M. A. M. Ito K. Hofmann S. (2020). Impact of culture medium on cellular interactions in *in vitro* Co-culture systems. Front. Bioeng. Biotechnol. 8, 911. 10.3389/fbioe.2020.00911 32850750 PMC7417654

[B58] Vriezen N. Romein B. Luyben K. C. van Dijken J. P. (1997). Effects of glutamine supply on growth and metabolism of mammalian cells in chemostat culture. Biotechnol. Bioeng. 54 (3), 272–286. 10.1002/(SICI)1097-0290(19970505)54:3<272::AID-BIT8>3.0.CO;2-C 18634093

[B59] Wang L. Li J. Guo L. Li P. Zhao Z. Zhou H. (2018). Molecular link between glucose and glutamine consumption in cancer cells mediated by CtBP and SIRT4. Oncogenesis 7 (3), 26. 10.1038/s41389-018-0036-8 29540733 PMC5852974

[B60] Wang Y. Wondisford F. E. Song C. Zhang T. Su X. (2020). Metabolic flux analysis—linking isotope labeling and metabolic fluxes. Metabolites 10 (11), 447. 10.3390/metabo10110447 33172051 PMC7694648

[B61] Washington I. M. Van Hoosier G. (2012). Clinical biochemistry and hematology. Laboratory Rabbit, Guin. Pig, Hamster, Other Rodents 2012, 57–116. Elsevier. 10.1016/B978-0-12-380920-9.00003-1

[B62] Waterval W. A. H. Scheijen J. L. J. M. Ortmans-Ploemen M. M. J. C. Habets-van der Poel C. D. Bierau J. (2009). Quantitative UPLC-MS/MS analysis of underivatised amino acids in body fluids is a reliable tool for the diagnosis and follow-up of patients with inborn errors of metabolism. Clin. Chim. Acta 407 (1–2), 36–42. 10.1016/j.cca.2009.06.023 19559691

[B63] Weidemann A. Lovas A. Rauch A. Andreas N. von Maltzahn J. Riemann M. (2016). Classical and alternative NF-κB signaling cooperate in regulating adipocyte differentiation and function. Int. J. Obes. 40 (3), 452–459. 10.1038/ijo.2015.198 26403432

[B64] Yu Y. Newman H. Shen L. Sharma D. Hu G. Mirando A. J. (2019). Glutamine metabolism regulates proliferation and lineage allocation in skeletal stem cells. Cell Metab. 29 (4), 966–978.e4. 10.1016/j.cmet.2019.01.016 30773468 PMC7062112

[B65] Zagari F. Jordan M. Stettler M. Broly H. Wurm F. M. (2013). Lactate metabolism shift in CHO cell culture: The role of mitochondrial oxidative activity. New Biotechnol. 30 (2), 238–245. 10.1016/j.nbt.2012.05.021 22683938

[B66] Zhang J. Pavlova N. N. Thompson C. B. (2017). Cancer cell metabolism: The essential role of the nonessential amino acid, glutamine. EMBO J. 36 (10), 1302–1315. 10.15252/embj.201696151 28420743 PMC5430235

