# Supplementary figures and images for: Non-ammoniagenic proliferation and differentiation media for cultivated adipose tissue
Source: Front Bioeng Biotechnol. 2023 Jul 24;11:1202165. doi: 10.3389/fbioe.2023.1202165 (PMC10405928; doi:10.3389/fbioe.2023.1202165)

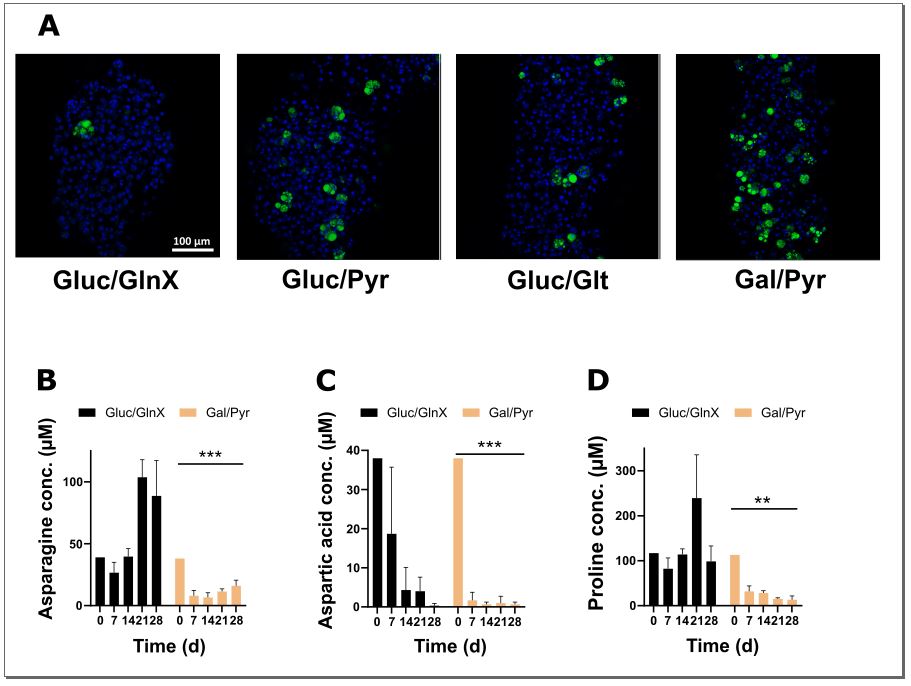

Supplement: Supplementary file 1 [file Image3.JPEG]

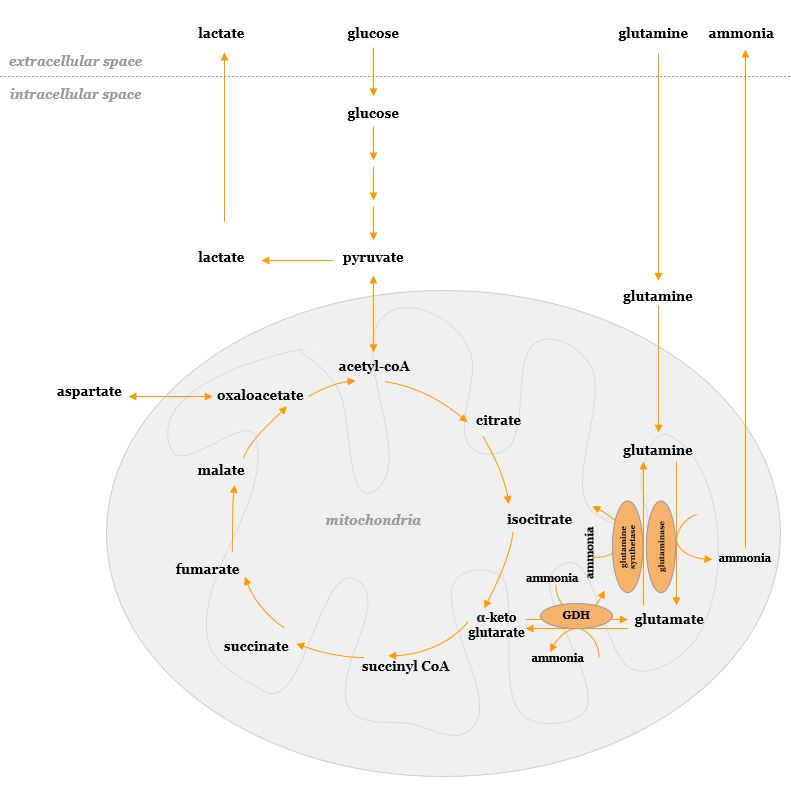

Supplement: Supplementary file 3 [file Image1.JPEG]

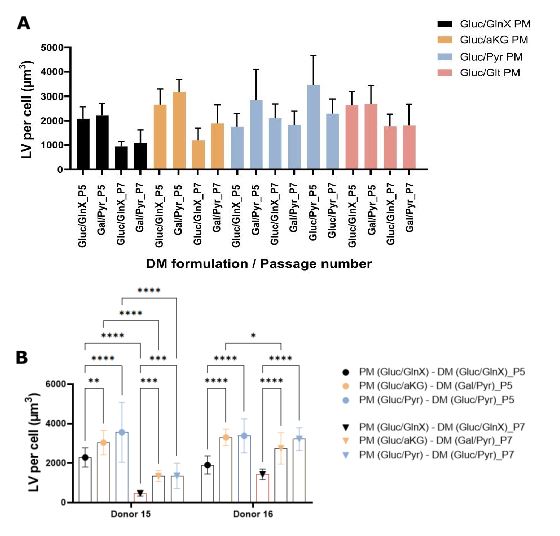

Supplement: Supplementary file 4 [file Image4.JPEG]

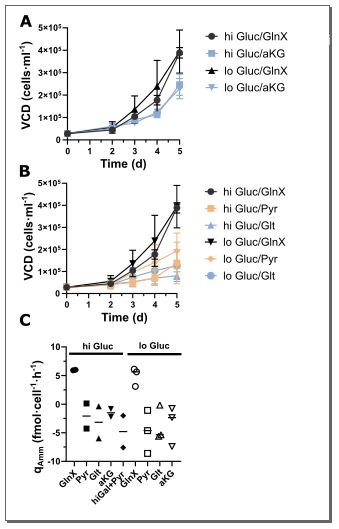

Supplement: Supplementary file 5 [file Image2.JPEG]

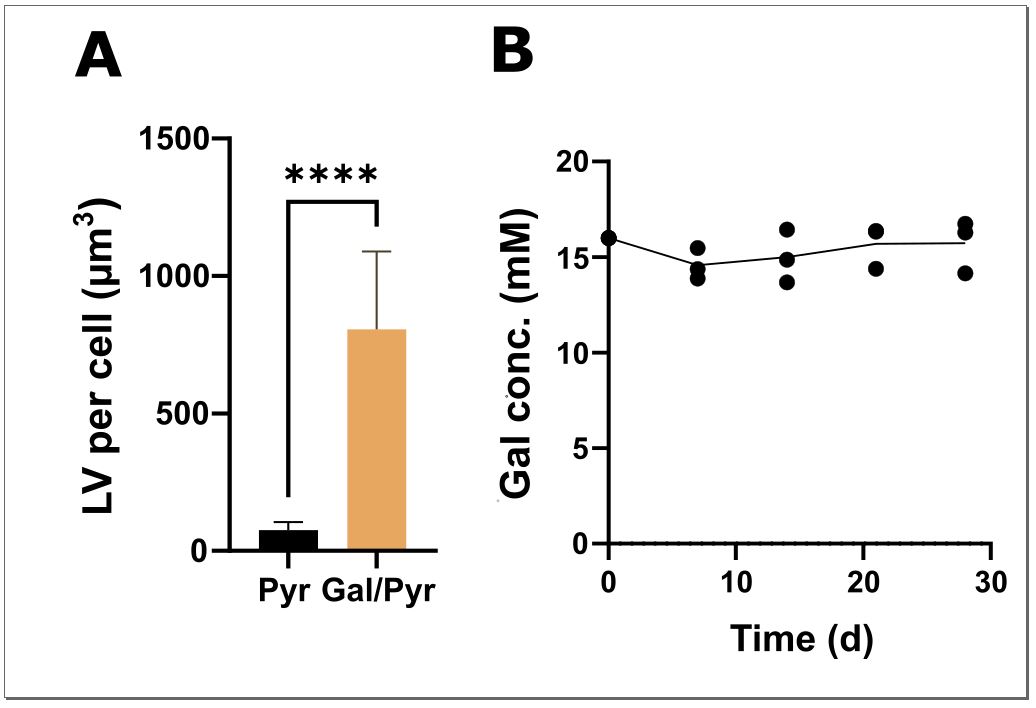

Supplement: Supplementary file 6 [file Image5.JPEG]
