# Supplementary material for: Non-ammoniagenic proliferation and differentiation media for cultivated adipose tissue
Source: Front Bioeng Biotechnol. 2023 Jul 24;11:1202165. doi: 10.3389/fbioe.2023.1202165 (PMC10405928; doi:10.3389/fbioe.2023.1202165)
Supplement: Supplementary file 2 [file Table1.DOCX]

**Table S1:** Control DM formulation

| **Component** | **Conc.** |
| --- | --- |
| **DM base** | |
| DMEM (12320032, Gibco™) |  |
| Chemically-defined FBS replacement | 1% |
| PSA (15140122, Gibco™) | 1% |
| **DM inducers** | |
| T3 (T6397-100MG, Sigma Aldrich) | 1 nM |
| Indomethacin (I7378, Sigma Aldrich) | 50 µM |
| Insulin (10-365, Peprotech) | 10 µM |
