# Supplementary material for: Non-ammoniagenic proliferation and differentiation media for cultivated adipose tissue
Source: Front Bioeng Biotechnol. 2023 Jul 24;11:1202165. doi: 10.3389/fbioe.2023.1202165 (PMC10405928; doi:10.3389/fbioe.2023.1202165)
Supplement: Supplementary file 7 [file Table2.DOCX]

**Table S2:** Full data set on long term passaging and differentiation in GlnX versus GlnX-replacements aKG, Pyr, Gal.

| **PM** | **DM** | **Donor** | **Passage** | **Mean LV per cell (μm^3^)** | **SD** | **n** |
| --- | --- | --- | --- | --- | --- | --- |
| Gluc/GlnX | Gluc/GlnX | 1 | 5 | 2286.0 | 484.4 | 25 |
|  | Gluc/GlnX | 2 | 5 | 1897.0 | 459.0 | 25 |
|  | Gal/Pyr | 1 | 5 | 2301.8 | 394.2 | 25 |
|  | Gal/Pyr | 2 | 5 | 2110.6 | 595.0 | 25 |
|  | Gluc/GlnX | 1 | 7 | 464.1 | 143.1 | 25 |
|  | Gluc/GlnX | 2 | 7 | 1424.7 | 264.3 | 25 |
|  | Gal/Pyr | 1 | 7 | 291.3 | 241.8 | 25 |
|  | Gal/Pyr | 2 | 7 | 1885.8 | 815.7 | 25 |
| Gluc/aKG | Gluc/GlnX | 1 | 5 | 1444.4 | 654.4 | 25 |
|  | Gluc/GlnX | 2 | 5 | 3880.1 | 613.6 | 25 |
|  | Gal/Pyr | 1 | 5 | 3036.0 | 619.6 | 25 |
|  | Gal/Pyr | 2 | 5 | 3307.0 | 414.7 | 25 |
|  | Gluc/GlnX | 1 | 7 | 507.1 | 386.3 | 25 |
|  | Gluc/GlnX | 2 | 7 | 1900.9 | 597.4 | 25 |
|  | Gal/Pyr | 1 | 7 | 1046.8 | 714.3 | 25 |
|  | Gal/Pyr | 2 | 7 | 2744.7 | 798.9 | 25 |
| Gluc/Pyr | Gluc/GlnX | 1 | 5 | 1971.8 | 610.3 | 25 |
|  | Gluc/GlnX | 2 | 5 | 1541.3 | 468.0 | 25 |
|  | Gal/Pyr | 1 | 5 | 3207.7 | 196.7 | 25 |
|  | Gal/Pyr | 2 | 5 | 2488.4 | 536.0 | 25 |
|  | Gluc/GlnX | 1 | 7 | 1472.0 | 567.7 | 25 |
|  | Gluc/GlnX | 2 | 7 | 2756.8 | 572.0 | 25 |
|  | Gal/Pyr | 1 | 7 | 1051.6 | 490.4 | 25 |
|  | Gal/Pyr | 2 | 7 | 2602.2 | 635.2 | 25 |
|  | Gluc/Pyr | 1 | 5 | 3559.4 | 1515.1 | 25 |
|  | Gluc/Pyr | 2 | 5 | 3378.8 | 859.1 | 25 |
|  | Gluc/Pyr | 1 | 7 | 1348.0 | 643.0 | 25 |
|  | Gluc/Pyr | 2 | 7 | 3217.4 | 576.4 | 25 |
| Gluc/Glt | Gluc/GlnX | 1 | 5 | 3109.2 | 782.0 | 25 |
|  | Gluc/GlnX | 2 | 5 | 2175.6 | 304.4 | 25 |
|  | Gal/Pyr | 1 | 5 | 3201.4 | 843.0 | 25 |
|  | Gal/Pyr | 2 | 5 | 2160.9 | 671.8 | 25 |
|  | Gluc/GlnX | 1 | 7 | 1291.1 | 445.6 | 25 |
|  | Gluc/GlnX | 2 | 7 | 2243.8 | 537.4 | 25 |
|  | Gal/Pyr | 1 | 7 | 1363.2 | 444.9 | 25 |
|  | Gal/Pyr | 2 | 7 | 2247.3 | 1295.4 | 25 |
